# Study on a High-Current CNT Cathode X-Ray Tube

**DOI:** 10.3390/nano16090560

**Published:** 2026-05-02

**Authors:** Huaping Tang, Jinmei Chen, Guoyu Li, Sheng Lai, Wu He, Zhiqiang Chen

**Affiliations:** 1 Department of Engineering Physics, Tsinghua University, Beijing 100084, China; thp21@mails.tsinghua.edu.cn; 2 NuRay Technology Co., Ltd., Changzhou 213200, China; chenjm@nuraytech.com (J.C.); ligy@nuraytech.com (G.L.); lais@nuraytech.com (S.L.); hew@nuraytech.com (W.H.)

**Keywords:** carbon nanotubes, field emission, emission current density, current intensity, X-ray tube

## Abstract

This work aims to achieve both high current emission density and high emission current of carbon nanotube (CNT) cathodes for high-power X-ray generation applications. High-purity small-diameter CNT materials were obtained, and a novel “five-state” electrophoretic deposition method was proposed to fabricate CNT cathodes. For an emission area of 10 mm × 0.45 mm, a high and stable cathode emission current of 350 mA was achieved, corresponding to an emission current density of 7.8 A/cm^2^. An X-ray dose rate of 39.49 mGy/s@50 cm was measured under a tube potential of 120 kV, cathode current of 100 mA, and pulse width of 10 ms. The focal spot size of the X-ray source, measured using a slit camera, was 0.98 mm (width) × 1.05 mm (length) at 15% max intensity, and the pulse width range was 100 µs–100 ms. Through continuous testing at 200 mA emission current, 100 µs pulse width, and 0.3% duty cycle for 400 h, the CNT cathode is estimated to exhibit a lifetime of approximately 5085 h, demonstrating stable and reliable durability. This study, for the first time, simultaneously realizes multi-A/cm^2^-level emission current density, hundreds-of-milliampere emission current, and hundreds-of-millisecond operating pulse width for CNT cathodes.

## 1. Introduction

Carbon nanotubes (CNTs) were discovered in 1991 by the Japanese scientist Sumio Iijima [1]. Extensive studies have demonstrated that CNTs possess excellent properties, including high chemical stability, superior mechanical strength, and outstanding electrical and thermal conductivity. Moreover, owing to their unique geometric characteristics such as nanoscale tip curvature radius, large aspect ratio, and low electron work function, combined with exceptional electronic properties, CNTs are regarded as highly promising field emission materials. In 1995, Heer et al. [2] first investigated the field emission characteristics of CNTs and proposed the concept of using CNTs as electron-emitting cathodes, thereby initiating the research direction of CNT-based field emission cathodes. Shortly thereafter, CNT cathodes exhibited great potential as electron sources for X-ray generation [3,4,5], attracting widespread research interest.

In the early stage, CNT cathode X-ray tubes operated only at microampere-level currents. From 2002, researchers at the University of North Carolina at Chapel Hill (UNC) [6,7,8] reported significant progress in CNT cathode X-ray tubes, achieving pulsed emission currents of 28 mA, elevating the CNT cathode emission current from the microampere to the milliampere level and marking the beginning of their practical application potential. A large body of research has demonstrated that CNT cathode X-ray sources exhibit significant characteristics and advantages compared with conventional thermionic X-ray sources based on tungsten filaments [9], including: (1) The emitter operates at room temperature. Unlike traditional tungsten filament thermionic cathodes, it is a “cold cathode” that requires no preheating and consumes no filament power, offering clear energy-saving advantages in mobile or field applications; (2) CNT cathodes rely on field emission, typically controlled in a pulsed mode, enabling rapid transient switching. This feature is beneficial in X-ray imaging applications for reducing unnecessary radiation dose, lowering noise, and improving image quality [10]; (3) Compared with tungsten filament cathodes, CNT cathodes do not require heating or thermal shielding structures, resulting in a more compact form factor. This facilitates array integration and enables the development of multi-beam X-ray sources [11], demonstrating strong technological advantages in emerging stationary three-dimensional imaging applications [12].

In recent years, with advances in nanomaterial synthesis, structural design, and micro-/nano-fabrication technologies, the field emission performance of CNT cathodes has been substantially improved. CNT-based X-ray sources have achieved stable operation at anode currents of around ten milliamperes [13,14], reaching the threshold for practical applications such as security inspection and industrial non-destructive testing. Gonzales et al. [12] developed a security inspection stationary CT system based on a multi-beam X-ray source with carbon nanotube (CNT) cold cathodes, operating at an anode voltage of 160 kV and an anode current of 7 mA. Tang et al. [15] developed a CNT cold cathode multi-beam X-ray source for tomographic inspection of industrial products, achieving an anode voltage of 160 kV and an anode current of 15 mA. For medical applications, Tang et al. [10] implemented high-resolution dental tomographic imaging using a CNT X-ray tube operating at an anode voltage of 70 kV and an anode current of 7 mA. At the University of North Carolina, Billingsley et al. [16] conducted research on a stationary digital chest tomosynthesis using a 63-beam CNT cathode X-ray source developed by Tang et al. [17] at NuRay. With a pulsed anode current of 20.4 mA at 120 kVp, the feasibility of cold cathode X-ray sources for chest tomosynthesis imaging was demonstrated. However, in current clinical practice, conventional digital radiography (DR) typically requires X-ray tube anode currents of 100–500 mA [18], while medical CT systems generally operate at 150–400 mA [19], with high-end CT scanners reaching up to 1000 mA. Consequently, the operating current capability of existing CNT cathode X-ray tubes remains insufficient for medical applications.

Considerable efforts have been devoted to increasing the emission current of CNT cathodes. Leberl et al. [20] from Siemens AG achieved a maximum cathode current of 126 mA using pulsed emission with a pulse width of 200 µs; however, the emission area was as large as 62.5 mm^2^, resulting in a low emission current density of only 0.2 A/cm^2^. Northern Illinois University [21] employed a CNT cathode with a diameter of 15 mm and realized a pulsed emission current of 650 mA at a pulse width of 70 ps, corresponding to an emission current density of approximately 0.1 A/cm^2^. The Electronics and Telecommunications Research Institute (ETRI) of Korea [22] fabricated a multi-cathode distributed X-ray tube using CNT paste, with a single-cathode emission area of 74.1 mm^2^, achieving an emission current of 200 mA at an anode voltage of 80 kV and a pulse width of 100 µs, but with a low emission current density of only 0.27 A/cm^2^. Although these studies demonstrated emission currents on the order of several hundred milliamperes, the low current density necessitated large cathode areas [23].

However, in medical imaging, the X-ray focal spot size must not be too large and is typically on the order of about 1 mm^2^. An excessively large cathode emission area is difficult to focus into a small focal spot; therefore, it is necessary to increase the emission current density while reducing the cathode emission area. In studies aimed at enhancing emission current density, Rice University [24] achieved an emission current density of 5.8 × 10^3^ A/cm^2^ using CNT fibers with a diameter of 9 µm, but the total cathode current was only 3.6 mA. Korea University [25] further increased the emission current density to nearly 10^5^ A/cm^2^ using a tip of CNT film; however, due to the small emission area, the maximum emission current was limited to 22.4 mA. These results indicate that CNT cathode research has not yet achieved the simultaneous realization of high emission current density and high emission current. Although electron beam focusing can reduce the focal spot size, for target focal spot sizes below 1.0 mm^2^, the cathode emission area generally should not exceed 10 mm^2^. Therefore, it is necessary to further investigate technologies and methods that can simultaneously increase the emission current density of CNT cathodes to several A/cm^2^ while achieving emission currents of several hundred milliamperes.

In research targeting both high emission current density and high emission current. Researchers from Xintek, Inc. reported a pulsed cathode emission current of over 1 A at 10 μs pulse width from a 2 × 15 mm CNT cathode (current density of ~3.3 A/cm^2^) [26]. Chen et al. [27] from the University of Electronic Science and Technology of China employed a large number of micro-scale CNT emission units arranged in an array, achieving a relatively high emission current density of 3.55 A/cm^2^. A pulse emission current of 710 mA was obtained at a pulse width of 8.5 µs. However, the pulse duration was short, and the cathode was found to be prone to damage, failing to meet the stability requirements for practical applications. Han et al. [28] from Korea University fabricated a CNT cathode electron source using a CNT mesh film technique, achieving an emission current density of 152 A/cm^2^ and a cathode current of 110 mA at a pulse width of 100 µs. Nevertheless, the stability was tested for only 100,000 pulses, and the pulse duration remained short, far below the hundreds of thousands of seconds required for medical applications. Overall, CNT-cathode X-ray tubes have not yet demonstrated high-current operation at pulse widths on the order of hundreds of milliseconds. Moreover, issues such as operational lifetime under high emission current density and currents exceeding 100 mA, pulse-to-pulse repeatability, reliability and stability during continuous operation, and other related factors still require in-depth investigation.

To overcome the limitations of long-term stability and reliability under high-current operation, this study is based on the preparation of high-purity CNTs of a specific type. Through material composition optimization and ultra-purification techniques, high-purity small-diameter CNTs with superior field emission performance were obtained. A five-state electrophoretic deposition technique was further proposed for cathode fabrication, resulting in a high-performance CNT cathode with an emission current density of 7.8 A/cm^2^ and a cathode emission current of 350 mA. The fabricated cathode was assembled into an experimental X-ray tube and evaluated under practical operating conditions, including an anode voltage of 120 kV, a cathode current of 100 mA, and a pulse width of 10 ms, with focal spot size and other parameters tested. In addition, a high-current lifetime test comprising 4.32 × 10^7^ pulses was conducted at a cathode emission current of 200 mA, an anode operating current of 120 mA, and a pulse width of 100 µs. The results demonstrate that the developed CNT cathode X-ray source achieves low-threshold voltage operation, high emission current, excellent stability, and long operational lifetime, thereby breaking through the technical bottleneck of hundred-milliampere-level anode current and highlighting the strong potential of CNT cathode X-ray sources for medical applications.

## 2. Methods and Experimental

**Figure 1:**
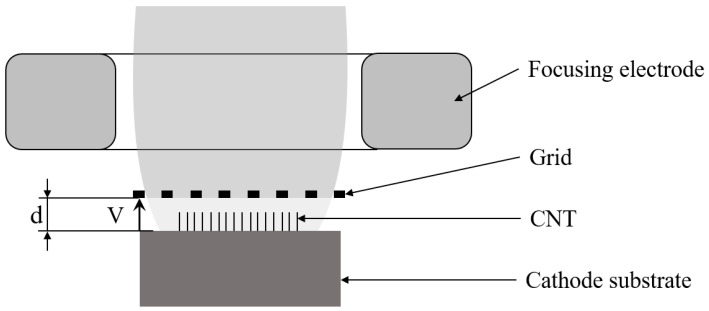
Schematic diagram of the CNT cathode structure.

CNT cathodes commonly adopt a cathode–grid structure, as schematically illustrated in Figure 1. In this configuration, CNT material is attached to the surface of a metal substrate and located beneath a grid mesh. By applying a grid voltage between the cathode and the grid, an electric field is generated, which lowers the surface potential barrier of the cathode material and narrows the barrier width. As a result, electrons at the sharp tips of the CNTs tunnel through the potential barrier via the quantum tunneling effect and are emitted into the vacuum, forming the cathode emission current.

According to the Fowler–Nordheim (F–N) equation [29], the field emission current density of the cathode $J_{FN}$ can be expressed as follows:

(1) $$J_{FN}=a_{FN}\frac{F^{2}}{\emptyset }\exp\left(-\frac{b_{FN}\emptyset^{\frac{3}{2}}}{F}\right)$$

In the above equation, $a_{FN}\;and\;b_{FN}$ are referred to as the F-N field emission parameters 1 and 2, $a_{FN}\approx 1.5414\;[\mu A][eV]{\left[V\right]}^{-2}$, $b_{FN}\approx 6.83089{\left[eV\right]}^{-3/2}[V]{\left[nm\right]}^{-1}$. F denotes the electric field strength between the cathode and the grid electrode, and ∅ represents the work function of the CNT material, with ∅≈5 eV [30]. However, in practical experiments, the directly measured parameters are typically the cathode emission current and the grid voltage. Therefore, Equation (1) is usually rewritten into a more commonly used form:

(2) $$I_{c}=Sa_{FN}\frac{{\left(V/d\right)}^{2}}{\emptyset }\exp\left(-\frac{b_{FN}\emptyset^{\frac{3}{2}}}{V/d}\right)$$

In Equation (2), $I_{c}$ represents the cathode emission current, S is the effective emission area of the cathode, V is the voltage applied to the grid, and d is the distance between the cathode and the grid.

During experiments, to verify whether the cathode emission current originates from field emission behavior, the above equations must be employed for validation. However, the exponential form of Equation (1) presents significant difficulties for direct data analysis. In practical applications, this issue is commonly addressed by taking the logarithm of Equation (1), yielding the following expression:

(3) $$\ln\left(\frac{J}{F^{2}}\right)=-b_{FN}\emptyset^{\frac{3}{2}}\frac{1}{F}+\ln\left(\frac{a_{FN}}{\emptyset }\right)$$

In Equation (3), $a_{FN}$, $b_{FN}\;and\;\emptyset$ are constants, and $\ln\left(J/F^{2}\right)$ exhibits a linear relationship with 1/F. Therefore, the experimentally measured I-V data can be transformed into $\ln\left(J/F^{2}\right)\;and$ 1/*F*, and the resulting curve is plotted. This curve is referred to as the F–N plot. If the plotted curve exhibits a linear trend, it indicates that the cathode electron emission follows a field emission mechanism.

The field enhancement factor β is used to describe the amplification of the electric field at the cathode surface induced by CNTs. A larger field enhancement factor indicates that a higher emission current can be achieved under a lower applied electric field. Since a practical cold cathode surface consists of a large number of CNTs, the average field enhancement factor of the cold cathode surface can be calculated using the following equation:

(4) $$\beta =-b_{FN}\frac{\emptyset^{\frac{3}{2}}}{k}$$

Here, k is the slope obtained from fitting the F–N curve [31].

The emission current of a CNT cathode is influenced by multiple factors, including the field emission electric field strength F, the type of CNTs, the field enhancement factor β at the CNT tips, the tip density of the CNTs, the CNT emission area S, and the grid transparency. The electric field F between the cathode and the grid can be adjusted through the grid-cathode gap d and the grid voltage V. Due to the practical feasibility of the grid-control circuitry and the requirement for precise tuning, the grid voltage V is typically limited to no more than 3 kV. To achieve a high electric field F, a smaller grid-cathode gap d is generally desired; however, d must be large enough to avoid grid shorting and electrical breakdown and is typically on the order of hundreds of micrometers.

The type and purity of CNT materials significantly affect emission performance, including the threshold field, emission current density, and stability, which depend on the CNT synthesis and purification techniques. The CNT tip density and tip field enhancement factor are interrelated and have a critical impact on both emission current density and cathode lifetime; both are determined by the cathode fabrication process. The CNT emission area and grid transparency also play important roles in determining the cathode current, and are closely related to the focal spot size, grid structure, and focusing design. Together with the grid-cathode gap d and other factors, these parameters constitute the main design considerations for the overall cathode structure.

### 2.1. Small-Diameter Carbon Nanotubes

The material properties of CNTs are key factors determining the performance of CNT cathodes, including CNT type, number of walls, diameter, aspect ratio, purity, and defect density. Studies have shown that CNTs with closed tips, compared to those with open tips, exhibit higher work function but better long-term operational stability [32]. Single-walled CNTs (SWCNTs) generally have low emission thresholds but are prone to structural damage, whereas multi-walled CNTs (MWCNTs) offer robust and reliable performance in field emission applications but have higher emission thresholds and are more difficult to control. Qian et al. [33] at Duke University and UNC developed a novel hybrid type of CNTs, few-walled CNTs (FWCNTs), by improving the synthesis of double-walled CNTs. FWCNTs consist of 2–5 coaxially rolled graphene layers, with diameters ranging from 2 to 10 nm, combining low field emission thresholds with good structural stability, making them ideal for achieving high emission current and long-term stability.

To realize CNT cathodes that can achieve both high emission current and long operational lifetime, the choice of CNT material is fundamental. In this work, high-purity, low-defect small-diameter CNTs (SDCNTs) with a certain number of layers were obtained, based on chemical vapor deposition (CVD) through process optimization and multi-step purification techniques, which was beneficial for obtaining a large field strength enhancement factor β. The procedure includes five main steps:

(1) Grow CNT materials with a high fraction of SDCNTs using an optimized combination of growth parameters;
(2) Remove carbonaceous impurities from the material via gas-phase oxidation at a controlled temperature;
(3) Select the regions with the best growth characteristics to extract high-purity SDCNTs;
(4) Eliminate catalyst metal ions through chemical treatment;
(5) Remove thermally and chemically resistant carbonaceous impurities via physical treatments to further purify the CNTs.

**Figure 2:**
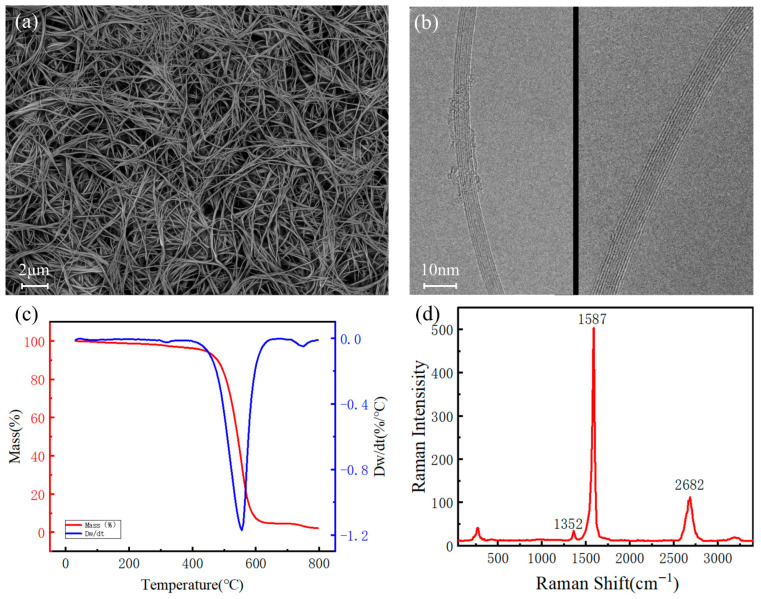
Images and thermogravimetric analysis of SDCNTs: (**a**) SEM, (**b**) TEM, (**c**) thermogravimetric analysis (TGA) results, and (**d**) Raman spectroscopy results.

The high-purity small-diameter CNTs prepared using this five-step process were characterized, and the results are shown in Figure 2.

Figure 2a shows the microstructure observed by scanning electron microscopy (SEM). The image reveals that the sample consists entirely of linear CNT structures, with minimal amorphous carbon or metallic impurity particles. The CNTs exhibit high uniformity and purity. Figure 2b presents the microstructure observed by transmission electron microscopy (TEM), showing two different CNT segments. It can be clearly seen that the small-diameter CNTs have a small number of layers, with smooth tube walls and well-defined interlayer boundaries, free of impurities or structural distortions. Moreover, with a diameter less than 10 nm, good field strength enhancement can be achieved in field emission. Figure 2c shows the thermogravimetric (TGA) curve of the CNT sample. The residual impurity content is less than 1%, and the decomposition temperature reaches 550 °C, indicating that the small-diameter CNTs possess highly stable chemical structures, strong interatomic bonding, high purity, and low defect density. Figure 2d presents the Raman spectrum obtained under a 532 nm excitation wavelength. The G peak at 1587 cm^−1^ is strong and sharp, indicating high crystallinity and well-ordered structure of the small-diameter CNTs. The D peak at 1352 cm^−1^, representing structural defects, is very weak, with an ID/IG ratio of only 0.06. This demonstrates an extremely low defect density and high degree of graphitization, suggesting that the small-diameter CNTs can fully realize their intrinsic performance advantages in cold-cathode X-ray source applications.

### 2.2. CNT Cathodes

The fundamental unit of a CNT cathode is the combination of CNTs with a conductive substrate, and there are several approaches to realizing this. These include: direct growth of CNTs on selected regions of a metal surface with a catalyst [27]; screen printing of CNT paste onto metal or conductive films [34]; electrophoretic deposition (EPD) in which a CNT solution is used and the metal substrate is immersed as an electrode [35,36]; mechanically attaching CNTs prepared in a specific shape to metal structures [25]; and cold pressing of CNT materials onto metal substrates [37]. Among these methods, EPD is characterized by simple equipment, a straightforward and easily controlled process, high reliability, and good reproducibility. To achieve both high emission current density and high total emission current, methods relying solely on high-density CNT fibers [24] or tips of CNT film [25] were not adopted. Instead, a CNT cathode with a moderate emission area was chosen. Methods such as direct growth and EPD allow precise control over the shape and area of the CNT cathode. To avoid the poor adhesion and short lifetime associated with directly grown CNTs on metal substrates [27], this study employed the EPD method. However, current CNT cathodes prepared via conventional EPD [38] exhibit limited emission current density, necessitating further optimization and improvement.

**Figure 3:**
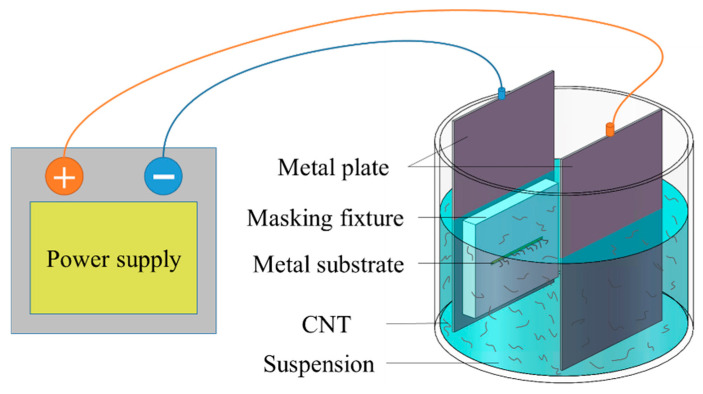
Schematic of the deposited state.

**Figure 4:**
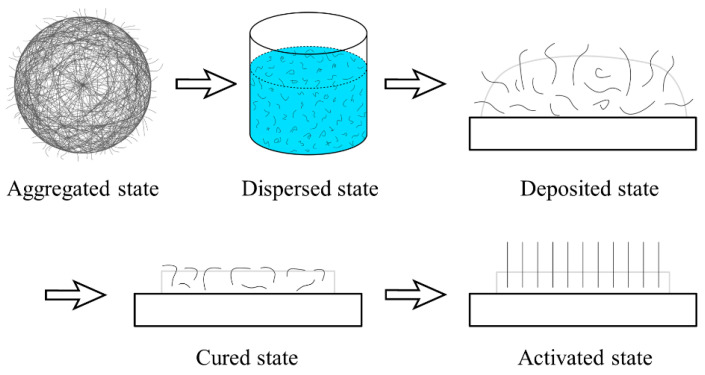
Five-state EPD technique.

In this work, the EPD process, as illustrated in Figure 3, was refined with the goal of substantially increasing the emission current density, with additional auxiliary steps and measures applied. A novel “five-state” EPD technique was developed, enabling CNT cathodes based on small metal blocks to achieve the following characteristics: high CNT density on the surface, uniform distribution, vertically aligned bundles, strong adhesion, and spatially patterned and controllable emission regions. This approach significantly enhances emission current density, achieving a balance between high emission current density and large cathode current. The five states of this process are: aggregated state, dispersed state, deposited state, cured state, and activated state, as illustrated in Figure 4.

**Figure 5:**
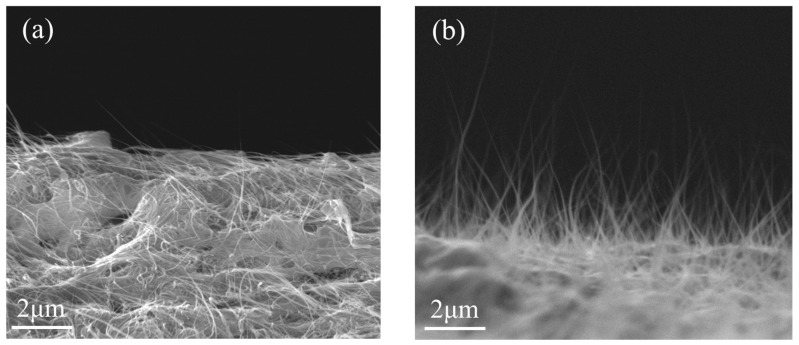
SEM images of the CNT morphology on the cold cathode surface before and after activation: (**a**) before activation, (**b**) after activation.

During the curing process, the added curing agent may partially cover or embed some CNTs, and most CNTs lie flat on the metal substrate surface (Figure 5a). This configuration results in a CNT cathode with a long lifetime but low emission current density. In this study, the cured cathode was further treated in a vacuum environment by ion bombardment aging, which caused exposed or shallow CNTs to fracture and form tips. Subsequently, high electric fields were applied (electrical annealing) to activate the CNT tips into an upright orientation (Figure 5b). The resulting CNTs are vertically aligned, well-organized, and uniformly distributed on the metal substrate, which is key to achieving a significant increase in cathode emission current density. This vertically well-aligned state of CNTs on the cathode surface is referred to as the activated state.

As shown in Figure 5b, after activation, a large portion of the CNTs on the cathode surface stand upright. The tips of these CNTs experience strong local electric field enhancement, allowing high emission currents at relatively high control voltages. Although the CNTs are densely distributed, they are not closely packed or aggregated, maintaining submicron spacing. This spacing effectively prevents electric field shielding between adjacent CNTs, ensuring that each CNT tip contributes to electron emission and enhancing the emission current density. Furthermore, the uniform CNT density across the entire cathode surface improves the overall emission performance.

Although a high electric field is directly applied to an as-cured CNT cathode or a conventional CNT film for activation, some CNTs can be induced to align vertically, but the number of CNT tips protruding from the film remains limited. This is because a large number of CNTs on the surface remain entangled with each other or have their tips buried beneath the surface. As a result, field activation alone cannot achieve the densely aligned, vertically oriented CNT configuration shown in Figure 5b, making it difficult to realize high emission current density.

The design of the CNT cathode emission area shape is also an important consideration. Once a high emission current density J is achieved using the above methods, the total cathode emission current $I_{C}$ is proportional to the emission area S, as expressed in Equation (5):

(5) $$I_{C}=J\times S$$

**Figure 6:**
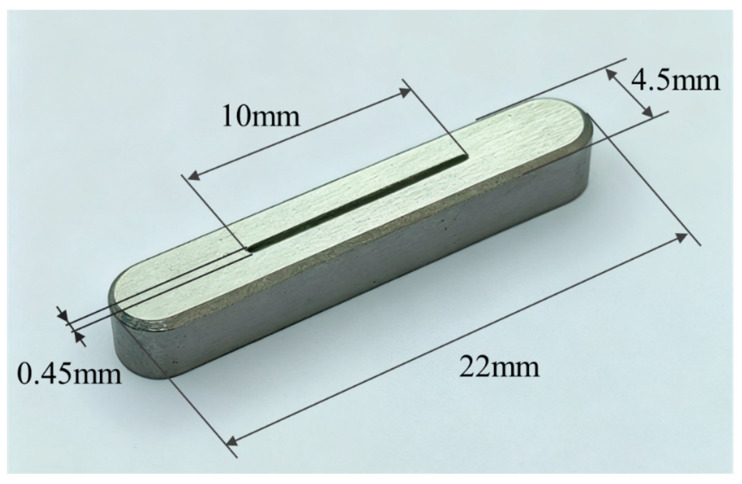
Photograph of the CNT cathode.

If the cathode adopts a regular shape, such as a rectangle, the emission area is the product of its length and width. In an X-ray tube, the focal spot size is the most direct factor determining the spatial resolution of X-ray imaging. In medical applications, to achieve high-resolution imaging, the focal spot size is generally required to be no larger than 1.2 mm, and in applications such as mammography, it may need to be as small as 0.3 mm [39]. In X-ray tube design, the effective focal spot size can be reduced using focusing techniques, with a typical achievable focusing ratio of approximately 5:1. Accordingly, in this study, the target X-ray focal spot size was set to 1 mm^2^, limiting the cathode emission area S to no more than 5 mm^2^. By applying the Line Focus Principle, a rectangular cathode emission region can produce a square projected focal spot. Based on this, the CNT cathode emission region was designed to be 10 mm × 0.45 mm, with the 10 mm length aligned along the reflection target projection direction, resulting in an emission area S of 4.5 mm^2^. The CNT cathode was then fabricated on the metal substrate using the five-state electrophoretic deposition method described above, as shown in Figure 6.

To enhance the performance of the CNT cathode in X-ray tube applications, the cathode underwent structural optimization, including the design of an appropriate grid and cathode-to-grid spacing d, as well as the implementation of a focusing structure. These designs allow the previously designed 4.5 mm^2^ CNT emission area to produce an appropriately sized X-ray focal spot through focusing. Additionally, to test and verify the CNT cathode’s emission current density, total emission current, and real operational characteristics in an X-ray tube, an open-type X-ray tube chamber was designed. The chamber includes an anode, X-ray window, flange, and vacuum port. The open flange design allows the cathode to be easily assembled and disassembled for repeated testing.

### 2.3. Cold Cathode Assembly Design

**Figure 7:**
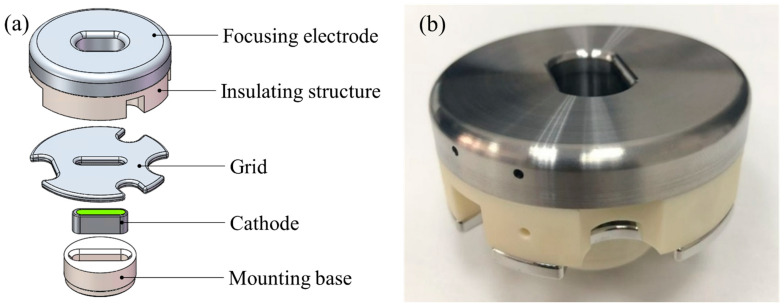
Structure of the CNT cathode assembly: (**a**) cathode component, (**b**) prepared cathode.

The performance of a cold cathode is significantly influenced by multiple components in its structure. The grid mesh directly determines the electron beam transmission rate and also affects beam quality and cathode lifetime. Its design must balance transmission efficiency and structural reliability. The cathode-to-grid spacing has a major impact on the required control voltage. As the spacing increases, the control voltage rises. Larger spacing facilitates machining and assembly but makes grid control more challenging, while smaller spacing demands higher machining and assembly precision and is more prone to arcing, which can drastically shorten cathode lifetime. Therefore, the spacing must be optimized considering machining precision, implementation cost, and cathode lifetime. The cold cathode assembly designed in this study is shown in Figure 7.

### 2.4. X-Ray Tube Chamber Design

**Figure 8:**
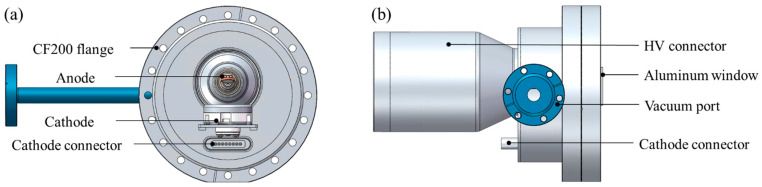
Structural design of the X-ray experimental tube: (**a**) front view (without front flange), (**b**) side view.

The X-ray tube chamber is mainly used to evaluate the electron emission and operational performance of the CNT cathode in an X-ray tube. For practical implementation, the chamber was designed according to conventional parameters. The anode high-voltage target parameter was set to 120 kV, and a fixed anode target configuration was adopted for ease of machining. The high-voltage connector uses a ceramic insulator and is designed to be compatible with a standard R24 high-voltage cable. The main body of the X-ray tube chamber is a circular stainless-steel housing with a CF200 standard circular flange. It adopts an open structure that can be opened from the front side to allow convenient installation and replacement of the CNT cathode and anode. The cathode and anode are mounted facing each other inside the tube. The anode uses a fixed reflective target structure with an anode angle of 16°. The tube chamber is also equipped with a cathode feedthrough for control signals, an aluminum X-ray window for X-ray output, and a vacuum port to maintain a high vacuum inside the tube. The detailed structural design is shown in Figure 8.

**Figure 9:**
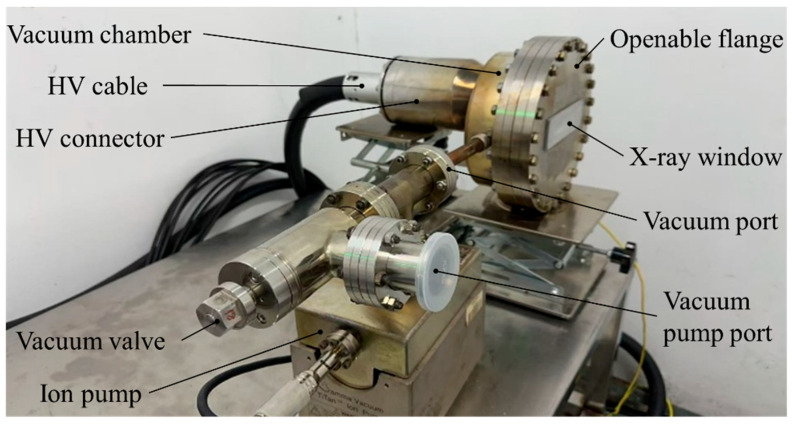
X-ray experimental tube.

According to the above design, the X-ray tube experimental chamber was fabricated and assembled, as shown in Figure 9. The experimental chamber is connected to the vacuum system through a vacuum port, including an ion pump, vacuum valve, and vacuum pumping unit (a combination of a mechanical pump and a turbo-molecular pump). After cathode installation, the sealed X-ray tube experimental chamber is heated and baked. First, the vacuum pumping unit is operated to achieve a vacuum level better than $10^{-4}$ Pa inside the chamber. The vacuum valve is then closed, and the ion pump is used to further increase the vacuum level to better than $10^{-6}$ Pa, maintaining the chamber in a high-vacuum state.

As shown in Figure 9, the X-ray experimental tube maintains an ultra-high vacuum inside the tube through continuous operation of the ion pump. A high-voltage cable is connected via the high-voltage connector to a Spellman ST120P12 high-voltage power supply (maximum output voltage: 120 kV, maximum current: 100 mA). The cathode interface is connected to a self-developed cathode control unit (NuRay, ECS-H032S, maximum output voltage: 3 kV, maximum current: 500 mA). In this configuration, the tube can operate over a wide parameter range: anode voltage from 10 kV to 120 kV, cathode current from 20 mA to 350 mA, and pulse width from 100 μs to 100 ms.

## 3. Results and Discussion

After assembling the X-ray experimental tube, heating and baking were performed and the tube was evacuated to an ultra-high vacuum better than $10^{-6}$ Pa. The anode voltage, cathode current, and pulse width were then gradually increased (“conditioning”) from low to high values. Once stable operation was achieved, tests were conducted on CNT cathode emission performance, X-ray tube operating parameters, maximum cathode current, and cathode lifetime. The measured current–voltage (I–V) characteristics were used to quantitatively analyze the field-emission behavior of the CNT cathode. Based on parameters such as anode voltage, anode current, and dose rate, the operating characteristics of the X-ray tube were evaluated. The practical operating state of the CNT cathode inside the X-ray tube was further used to assess its emission capability, operational stability, and service lifetime, thereby evaluating the applicability of this CNT-cathode X-ray tube in medical imaging.

### 3.1. CNT Cathode Emission Performance Test

Using the X-ray experimental tube shown in Figure 9, the CNT cathode was operated under constant-current control via a self-developed electronic control system (ECS). Specifically, the ECS adjusts its output control voltage according to the preset target current to stabilize the emission current. The maximum control voltage is limited to 3 kV, which prevents electrical breakdown or arcing between the cathode and the gate, thereby ensuring the safety and reliability of the emitter.

**Figure 10:**
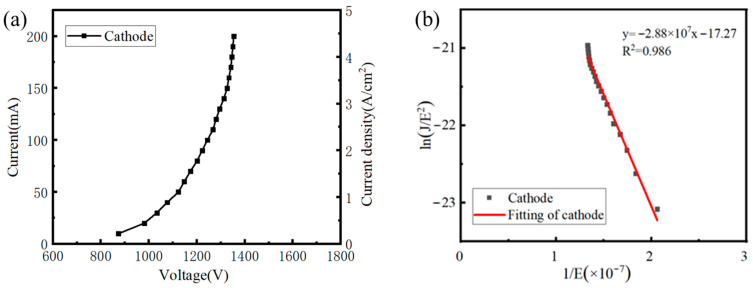
Field emission performance of CNT cathode: (**a**) current–voltage operating curve, (**b**) F–N plot.

At an anode voltage of 120 kV, pulse width of 10 ms, and duty cycle of 1%, the ECS output voltage was adjusted so that the cathode current increased stepwise from 10 mA to 200 mA in increments of 10 mA. The corresponding grid-control voltages for each cathode current were recorded to obtain the I–V (current–voltage) working curve of the CNT cathode. The test data were fitted using F–N theory to evaluate the field-emission characteristics of the CNT cathode and to calculate the field-enhancement factor by Equations (3) and (4), respectively. The test and analysis results are shown in Figure 10.

The test results show that at relatively low grid voltages, electron emission from the cathode is in an unstable operating state. As the voltage increases, the cathode surface attains sufficient field strength to enter a stable emission regime, and the emission current increases exponentially. From the I–V characteristic curve in Figure 10a, it can be seen that an emission current $I_{C}$ of 200 mA is achieved with a grid control voltage V of less than 1400 V. The corresponding emission current density J is approximately 4.44 A/cm^2^. The developed cathode therefore achieves both high emission current and high current density, indicating strong application potential in high-current, high-resolution imaging.

In the F–N fitting curve in Figure 10b, the coefficient of determination is $R^{2}=0.986$, indicating a very high degree of linear correlation. This demonstrates that the electron emission of the CNT cold cathode strictly follows the F–N tunneling mechanism, with no significant interference. During the test, the surface condition of the CNT cathode did not undergo drastic changes, and the uniformity and stability of the emission trends were good. From the fitted slope k = −2.89 × 10^7^, the field enhancement factor β of the cathode is calculated to be approximately 2642 using Equation (4). Compared with the existing high-current CNT cathode technology [20,21], the β value is larger in this work, indicating that the small-diameter CNT material used has enabled the cathode to achieve stronger performance. The intercept of the fitting line in Figure 10b is −17.3, which is consistent with −15 calculated by the intercept of ln($a_{FN}$/∅) in Equation (3).

**Figure 11:**
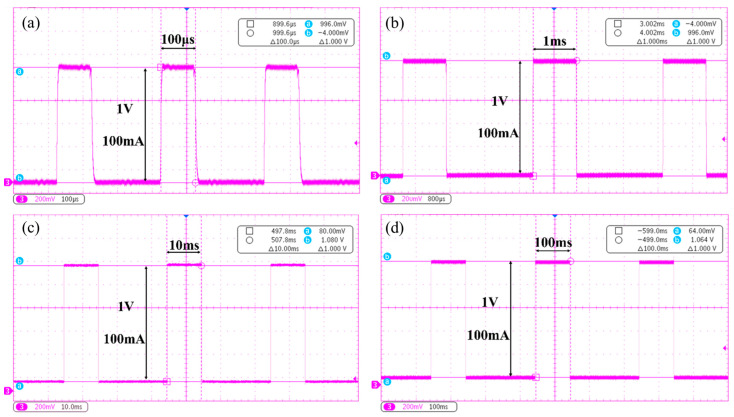
Operating performance of the CNT cathode at different pulse widths: (**a**) pulse width 100 µs, (**b**) pulse width 1 ms, (**c**) pulse width 10 ms, and (**d**) pulse width 100 ms.

By adjusting the ECS parameters, the operating performance of the CNT cathode under different pulse widths can be observed. Four operating modes with pulse widths of 100 µs, 1 ms, 10 ms, and 100 ms were tested. Considering the pulsed power tolerance of the anode target, the anode voltage was set to a relatively low value of 40 kV, and the cathode current was uniformly set to 100 mA. The test results are shown in Figure 11.

As shown in Figure 11, a Tektronix MDO3024 oscilloscope (Tektronix, Inc., Beaverton, OR, USA) was used to observe the emission current waveforms of the CNT cathode operating at an emission current of 100 mA under different pulse widths.

Figure 11a shows the waveform at a pulse width of 100 µs. The cathode current rapidly rises from 0 to 100 mA, with a rise/fall time of less than 5 µs, fully demonstrating the transient response characteristics of the CNT cathode.

Figure 11b shows the waveform at a pulse width of 1 ms, which has already taken on a standard square-wave shape, reflecting the precise constant-current control capability of the matching electronic control system.

Figure 11c shows the waveform at a pulse width of 10 ms. For the cathode current at the various pulse widths, no overshoot spike appears at the leading edge of the waveform, indicating that precise control of the cathode field-emission current can be achieved through accurate ramping of the grid voltage.

Figure 11d shows the waveform at a pulse width of 100 ms. The flatness at the top of the waveform is excellent. In this study, for the first time, output observation of a cathode current on the order of hundreds of milliamperes at pulse widths of hundreds of milliseconds was realized. Under these operating conditions, the cathode current maintained excellent stability, demonstrating that the CNT cathode is capable of operating at high current and long pulse width.

The anode current $I_{A}$ is the main parameter of the X-ray tube and is related to the cathode emission current $I_{C}$, the grid transmission coefficient $T_{g}$, and the anode capture efficiency p as follows:

(6) $$I_{A}=I_{C}\times T_{g}\times p$$

The grid transmission coefficient $T_{g}$ is an important performance parameter of the CNT cathode. In this study, a conventional strip-type grid design was adopted, and the width ratio of the grid strip (30 µm) to the grid spacing (70 µm) is 3:7. Therefore, the theoretical maximum transmission coefficient of the grid $T_{g}$ is 70%. The electron beam passing through the grid is not 100% captured by the anode but has a certain capture efficiency p. The product $T_{g}\cdot p$ can thus be referred to as the overall current efficiency.

**Figure 12:**
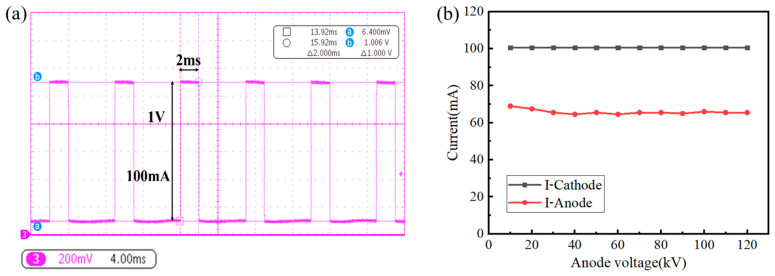
CNT cathode performance testing: (**a**) stability of cathode current between pulses, (**b**) anode current stability.

The operating stability of the X-ray tube cathode, as well as the stability of the anode current, is an important component of the stability analysis of the CNT cathode X-ray tube under pulsed operation. The X-ray tube was set to an anode voltage of 120 kV, a cathode current of 100 mA, and a pulse width of 2 ms, and the cathode current waveforms of multiple consecutive pulses were measured, as shown in Figure 12a, to analyze the cathode operating stability. With the cathode operating current fixed at 100 mA, the anode voltage was varied from 10 kV to 120 kV in steps of 10 kV. The average cathode current and average anode current for each measurement were recorded, and curves were plotted as shown in Figure 12b to analyze whether the overall current efficiency is affected by the X-ray tube anode voltage.

In Figure 12a, the cathode current of multiple consecutive pulses is 100 mA (10 mV corresponds to 1 mA). The variation in current between different pulses is better than 1%, showing excellent consistency. The specially developed electronic control system for the CNT cathode achieves very high current control accuracy, and the CNT cathode exhibits very good repeatability and stability. In Figure 12b, as the anode voltage gradually increases from 10 kV to 120 kV, the cathode current is independently controlled by the ECS and is consistently maintained at 100 mA, while the anode current changes slightly. According to Equation (6), the overall current efficiency $T_{g}\cdot p$ decreases slightly from 68.7% to 65.2%. Our analysis suggests that the change in anode voltage has very little effect on the grid transmission coefficient $T_{g}$. However, as the anode voltage increases, the yield of secondary electrons increases, and the focusing electric field generated between the focusing electrode and the anode differs slightly at different high anode voltages. This leads to a slight change in the capture efficiency p. Therefore, it is considered in this study that the slight decrease in the overall current efficiency is mainly caused by a slight reduction in the capture efficiency p.

### 3.2. Measurement of X-Ray Tube Operating Parameters

**Figure 13:**
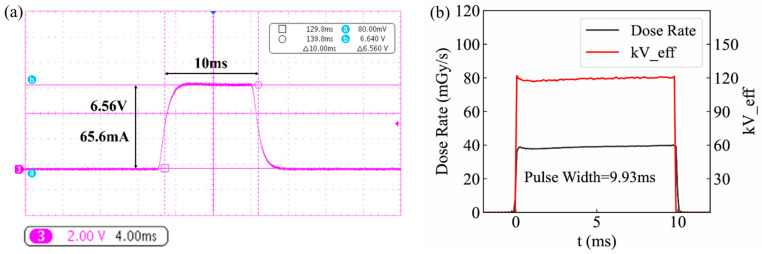
X-ray tube operating parameter test results: (**a**) anode current waveform, and (**b**) pulse dose rate and equivalent tube voltage waveform.

The anode voltage, anode current, and focal spot size are the three main operating parameters of an X-ray tube. In the CNT cathode experimental X-ray tube developed in this study, a simple fixed anode target structure was adopted, resulting in a relatively low target power capacity. Therefore, during high-voltage and high-current anode tests, the maximum pulse width was not used. For the experimental X-ray tube, the anode voltage was set to 120 kV, the cathode current to 100 mA, and the pulse width to 10 ms. Under X-ray emission conditions, a Tektronix MDO3024 oscilloscope was used to monitor the anode current signal from the high-voltage power supply, and the result is shown in Figure 13a. Meanwhile, a Radcal Accu-Gold AGMS-D+ X-ray analyzer (Radcal, Monrovia, CA, USA) was employed to measure the generated X-rays at a distance of 50 cm in front of the focal spot, and the result is shown in Figure 13b.

As shown in Figure 13a, for the CNT cathode experimental X-ray tube, when the cathode current was set to 100 mA, the measured anode current was 65 mA (1 V corresponds to 10 mA). According to Equation (6), the product of the electron transmission coefficient and capture efficiency is approximately 65%. Figure 13b shows that the X-ray pulse width is 9.93 ms, with the dose turning on and off almost instantaneously. The dose rate waveform closely approximates a standard square wave, demonstrating the excellent transient response of the CNT cathode. At a distance of 50 cm from the focal spot, the measured dose rate was 39.49 mGy/s. During pulsed operation of the X-ray tube, the anode voltage remained stable at 120 kV without fluctuations due to pulse initiation or termination. The experimentally measured X-ray parameters of the tube obtained with the Radcal analyzer are summarized in Table 1. It can be seen that the actual operating parameters of the experimental X-ray tube are in good agreement with the set values.

**Figure 14:**
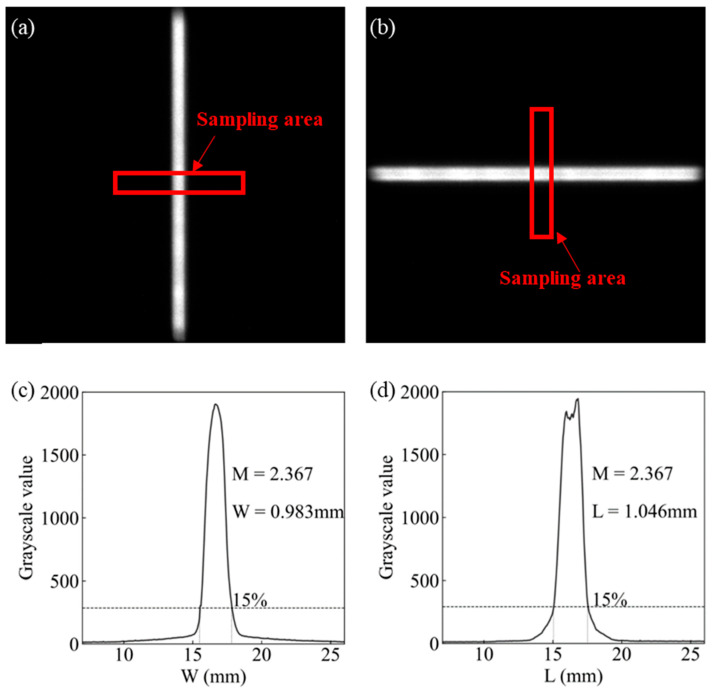
Focal spot measurement: (**a**) slit image in the width direction, (**b**) slit image in the length direction, (**c**) grayscale distribution along the width, and (**d**) grayscale distribution along the length.

The focal spot size is a critical parameter of an X-ray tube, as it plays a decisive role in determining the spatial resolution of medical imaging. In this study, achieving both high emission current density and high cathode current was particularly important in order to reduce the cathode emission area and thus obtain a small focal spot. To measure the actual focal spot size of the CNT cathode with an emission area of 10 mm × 0.45 mm, after applying the focusing structure and the line-focus effect of the reflective anode target, a slit-camera method was employed. The measurement was performed in accordance with IEC 60336:2020 [40]. The measured focal spot is shown in Figure 14.

Figure 14a,b present the focal spot images obtained on the detector after X-rays from the experimental X-ray tube passed through the slit. These correspond to the focal spot width and length, respectively. The slit was positioned between the X-ray window and the flat-panel detector, with an imaging magnification of M=2.367. In Figure 14a, the grayscale values of multiple rows of detector pixels in the central region of the focal spot (perpendicular to the light band) are sampled to generate the profile shown in Figure 14c. In Figure 14b, the grayscale values of multiple columns of detector pixels in the central region (also perpendicular to the light band) are sampled and averaged to produce the curve shown in Figure 14d. For each profile, the effective focal spot size is determined by taking the pixel distance corresponding to the width at 15% of the peak intensity and dividing it by the magnification factor M. The measured focal spot size is 0.98 mm (width) × 1.05 mm (length). This relatively small focal spot provides a solid foundation for achieving high spatial resolution and high image quality in X-ray imaging systems.

### 3.3. High Cathode Current Testing

**Figure 15:**
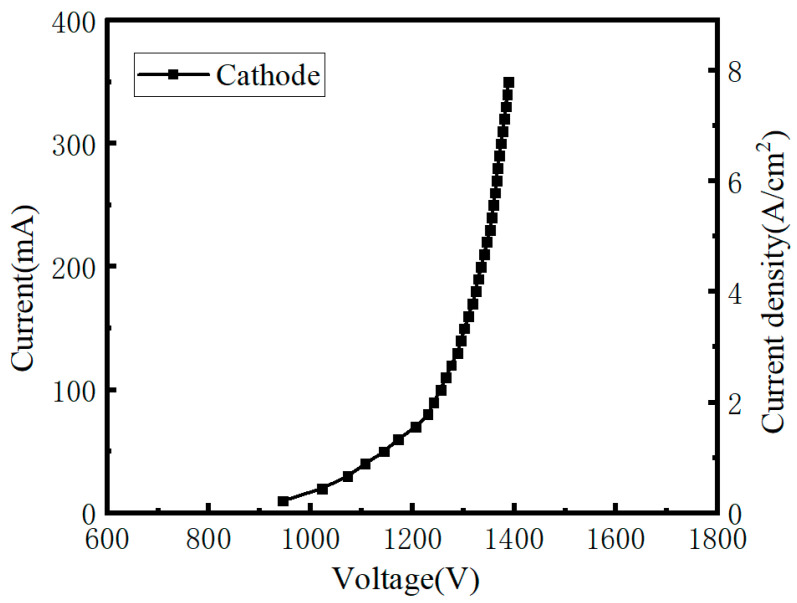
High-current testing of the CNT cathode.

The X-ray tube in this study uses a fixed anode target, and the anode power imposes a limitation on the cathode current. In the previous conditioning and testing, the CNT cathode operating current was limited to 200 mA; however, this does not represent the maximum current the CNT cathode can achieve. To evaluate the high-current performance of the CNT cathode, the anode voltage was reduced (while remaining within the anode power limit), allowing further testing of the CNT cathode at higher emission currents. The resulting I–V characteristics are shown in Figure 15.

The test results indicate that, based on the high-purity small-diameter carbon nanotube material and the five-state fabrication method developed in this study, the CNT cathode can achieve a maximum emission current $I_{C}$ of 350 mA at a grid voltage *V* of 1388 V. The corresponding emission current density *J* is approximately 7.78 A/cm^2^, fully meeting the requirements of medical imaging systems. Under high-current operation, the slope of the F–N curve corresponding to Figure 15 is −4.67 × 10^7^ (value as fitted). Using Equation (4), the effective field enhancement factor is calculated to be β = 1726. This value is lower than that obtained under relatively low current operation (200 mA). This behavior is consistent with the physical mechanism that, as the cathode control voltage is further increased, more CNT emission tips are activated for electron emission, and each individual CNT tip contributes more electrons. As a result, the effective collective field enhancement factor β decreases.

In this study, the CNT cathode achieved simultaneously high emission current (350 mA) and high emission current density (7.78 A/cm^2^). This performance can be mainly attributed to the following factors: Firstly, by optimizing the CNT growth process, high-purity few-walled carbon nanotubes were obtained (see Section 2.1). These CNTs exhibit a favorable balance between strong tip-induced field enhancement and structural stability during field emission. Secondly, a specialized “five-state” electrophoretic deposition (EPD) technique was employed to fabricate the emitter (see Section 2.2). In particular, the activation process plays a critical role. Through ion bombardment aging, CNTs protruding from or located near the film surface are fractured to form sharp tips. Subsequently, under a high electric field (electrical annealing), the CNT tips are induced to align vertically. Compared with other techniques, this approach offers two key advantages: (1) Vertically aligned CNT tips facilitate stronger local electric field enhancement at the tip apex, enabling high-current emission; (2) Ion bombardment generates a larger number of CNT tips, significantly improving their density and uniformity (as shown in Figure 5b), thereby enabling high emission current density over a millimeter-scale area.

### 3.4. Cathode Lifetime Testing

**Figure 16:**
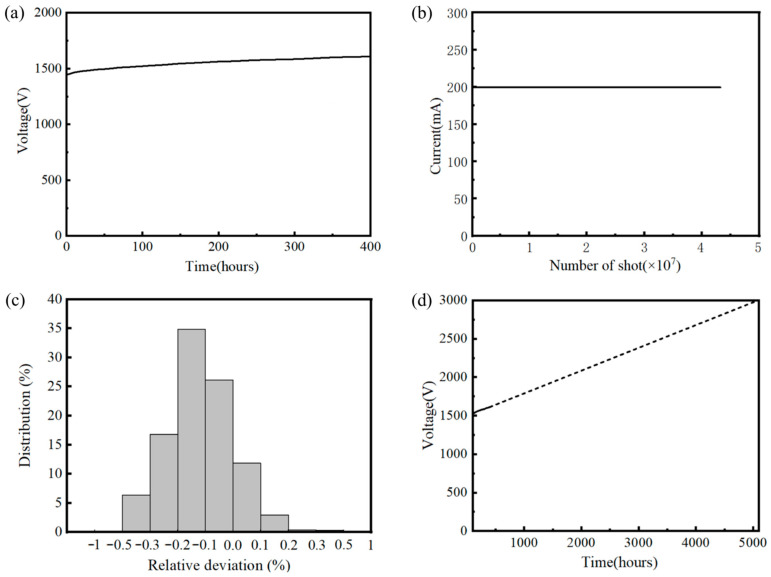
Long-term high-current performance testing of the CNT cathode: (**a**) grid voltage variation during long-term operation, (**b**) current stability during long-term operation, (**c**) cathode current precision during long-term operation, and (**d**) estimated lifetime analysis.

Lifetime is a critical performance parameter for the practical application of CNT cathodes. Compared with thermionic cathodes, which have benefited from over a century of optimization and technological development, CNT cathode technology is relatively new, has stricter environmental requirements, and its long-term lifetime has often been questioned. Existing studies on CNT cathode lifetime are limited, and the available data are insufficient to demonstrate long-term operation under high-current conditions [41]. To address this, long-term high-emission-current testing of the developed CNT cathode was performed using the aforementioned experimental X-ray tube under actual operating conditions. Due to the power limitation of the fixed anode target, relatively low anode voltage, pulse width, and duty cycle were used. The testing parameters were set as follows: anode voltage 40 kV, cathode operating current 200 mA, current pulse width 100 µs, and duty cycle 0.3%. The test results are shown in Figure 16.

The developed CNT cathode was tested at an emission current I_C_ of 200 mA with a pulse width T_w_ of 100 µs over a continuous duration of 400 h. The results show that, with a slight increase in the grid control voltage V (Figure 16a), the cathode maintained stable high-current emission of 200 mA over 4.32 × 10^7^ consecutive pulses (Figure 16b). During the test, under ECS control, fluctuations in the emission current were within 0.5% (Figure 16c). In this test, the initial grid control voltage V_a_ was 1443 V, and the corresponding increase in CNT cathode control voltage was 78 V, 41 V, 26 V, and 22 V respectively for every 100 h operating period, and the final grid voltage V_b_ at the end of testing was 1610 V. Although the grid voltage increased relatively rapidly at the initial stage of the lifetime test to maintain a constant current of 200 mA, the growth rate gradually slowed down in the later stage, demonstrating the operational stability of the CNT cathode. Using the designed maximum cathode control voltage V_max_ = 3000 V as the end-of-life criterion, and extrapolating based on the voltage growth slope observed during the latter 300 h, when the voltage evolution had entered a stable regime, the estimated operational lifetime of this CNT cathode at 200 mA, 100 µs pulse width, and 0.3% duty cycle is approximately 5085 h (Figure 16d). These results indicate that the developed CNT cathode has a reliable operational lifetime, capable of meeting practical application requirements and demonstrating strong potential for future deployment in relevant high-performance scenarios.

The CNT emitter developed in this study demonstrated an operational lifetime exceeding 5000 h at a working current of 200 mA with a pulse duty cycle of 0.3%. This performance can be attributed to the following factors: (1) A high density of vertically aligned CNTs on the cathode surface ensures that the emission current per individual CNT tip is relatively low, which is beneficial for long-term stability and extended lifetime; (2) The operating currents (200 mA or even 350 mA) remain well below the maximum emission capability of the emitter, and the emission is initiated at relatively low gate control voltages, both of which contribute to prolonged cathode lifetime; (3) A dedicated constant-current electronic control system (ECS), specifically developed for CNT cathodes, prevents overcurrent and electrical breakdown during operation, providing effective protection for the emitter; (4) During operation, the X-ray tube is maintained at a high vacuum level (better than 10^−6^ Pa) using an ion pump, which suppresses ion back-bombardment on the CNTs and thereby enhances emitter lifetime.

## 4. Conclusions

Based on high-purity small-diameter CNT fabrication technology and the “five-state” electrophoretic deposition method, a CNT cathode combining high current density J and high emission current *I_C_* was successfully developed. Tests in an X-ray tube demonstrated that, within a CNT emission area of 10 mm × 0.45 mm, the cathode can stably emit 350 mA, corresponding to an emission current density of 7.78 A/cm^2^, and is capable of continuous pulsed operation at 100 mA (2.22 A/cm^2^) with a long pulse width of 100 ms. This represents the first report of a CNT cathode simultaneously achieving multi-A/cm^2^-level current density, hundreds of mA emission current, and a hundred-millisecond pulse width. These results indicate that CNT cathodes can simultaneously realize small focal spots and large currents in X-ray tubes, particularly for X-ray imaging applications such as medical imaging. Compared with existing high-current technologies that require cathode emission areas on the order of several tens of mm^2^ [20,21,22], the emission area is reduced by approximately 90%; alternatively, compared with current small-focal-spot imaging applications limited to ~10 mA emission current [14,15,17], the emission current is increased by an order of magnitude. This work therefore demonstrates, for the first time, the practical application potential of CNT-based X-ray tubes in medical imaging and other fields with stringent performance requirements.

Furthermore, long-term testing at 200 mA emission current, 100 µs pulse width, and 0.3% duty cycle over 400 h indicates that this CNT cathode is projected to operate for approximately 5085 h. This result overcomes the long-standing reliability challenges of CNT cathodes under high-current operation. Compared with previously reported CNT cathode high-current tests limited to 10^5^ pulses [28] or lifetimes of only several tens of hours [26,27], the lifetime is improved by at least one order of magnitude. This reliable long-term stability paves the way for the commercial application of high-current CNT cathodes and represents a critical step toward the practical deployment of CNT-based X-ray tubes.

Further evaluation in an X-ray tube under operating conditions of 120 kV anode voltage, 100 mA cathode current, and 10 ms pulse width yielded an X-ray dose rate of 39.49 mGy/s at a distance of 0.5 m in the forward direction. The focal spot size, defined at 15% intensity width, is 0.98 mm (width) × 1.05 mm (length). These results demonstrate that the CNT cathode X-ray source fully meets the performance requirements for practical applications in industrial inspection, security screening, and medical imaging, and can achieve high-quality X-ray Imaging with a focal spot size of approximately 1 mm. This work provides critical technical support for the practical implementation of CNT cathode X-ray sources in application scenarios demanding high performance.

## Figures and Tables

**Table 1:** Radcal-measured X-ray parameters of the experimental tube.

Item	Parameter
Pulse width/ms	9.93
Dose rate/mGy/s	39.49
Aluminum half-value layer/mm	3.892
Tube voltage/kV	120.5

## Data Availability

All data used in this study are available upon request from the corresponding author.
